# Sleep Facilitates Extraction of Temporal Regularities With Varying Timescales

**DOI:** 10.3389/fnbeh.2022.847083

**Published:** 2022-03-25

**Authors:** Itamar Lerner, Mark A. Gluck

**Affiliations:** ^1^Department of Psychology, The University of Texas at San Antonio, San Antonio, TX, United States; ^2^Center of Molecular and Behavioral Neuroscience, Rutgers University, Newark, NJ, United States

**Keywords:** sleep, memory consolidation, extraction of regularities, insight, temporal scaffolding, pattern recognition

## Abstract

Evidence suggests that memory consolidation is facilitated by sleep, both through the strengthening of existing memories and by extracting regularities embedded in those memories. We previously observed that one sleep stage, Slow-Wave sleep (SWS), is particularly involved in the extraction of temporal regularities. We suggested that this attribute can naturally stem from the time-compressed memory replay known to occur in the hippocampus during SWS. A prediction coming out of this “temporal scaffolding” hypothesis is that sleep would be especially influential on extraction of temporal regularities when the time gap between the events constituting the regularities is shortish. In this study, we tested this prediction. Eighty-three participants performed a cognitive task in which hidden temporal regularities of varying time gaps were embedded. Detecting these regularities could significantly improve performance. Participants performed the task in two sessions with an interval filled with either wake or sleep in between. We found that sleep improved performance across all time gaps and that the longer the gap had been, the smaller was the improvement across both sleep and wake. No interaction between sleep and gap size was observed; however, unlike sleeping participants, awake participants did not exhibit any further performance improvement for the long gaps following the interval. In addition, across all participants, performance for the long gaps was associated with the development of conscious awareness to the regularities. We discuss these results in light of the temporal scaffolding hypothesis and suggest future directions to further elucidate the mechanisms involved.

## Introduction

A growing body of evidence from the last two decades suggests that sleep contributes to memory consolidation (Rasch and Born, 2013). Not only does sleep stabilize and strengthen recently acquired memories (e.g., Plihal and Born, 1997), but it also seems to be contributing to the generalization of these memories to new and partly similar circumstances by helping to extract the gist and identify patterns embedded within encoded experiences (Gomez et al., 2006; Djonlagic et al., 2009; Durrant et al., 2011; Lewis and Durrant, 2011; Lerner and Gluck, 2019; Lerner et al., 2019). This sleep-induced identification of patterns can be both explicit (e.g., Wagner et al., 2004; Fischer et al., 2006) and implicit (e.g., Ellenbogen et al., 2007; Lerner and Gluck, 2018), with particular sleep stages, such as Slow-Wave sleep (SWS) or Rapid-Eye-Movement (REM) sleep, preferentially contributing to specific types of generalization depending on the cognitive task used (Lerner and Gluck, 2019).

We recently argued that a particular type of sleep-induced extraction of patterns, namely, the identification of *temporal* regularities, could be a natural result of physiological processes occurring in the hippocampus during SWS (Lerner, 2017; Lerner and Gluck, 2019; Lerner et al., 2019). Temporal (or sequential) regularities are those in which an event happening at time *t* reliably predicts another event happening at time *t* + T. Such regularities, we argued, might be difficult to detect when experienced during wake if participants are uninformed of their possible existence, even if they are only seconds apart, because by the time the later event occurs, the first event is no longer active in working memory. Nevertheless, the series of events containing the regularity can still be stored in the hippocampus. Substantial evidence from animal studies suggests that during SWS, recently stored episodic memories are replayed in the hippocampus at an accelerated pace, a process that contributes to memory consolidation (Rasch and Born, 2013). Such time-compressed reenactment of the original sequences of events may bring the predictor and predicted events within Hebbian timescales (see August and Levy, 1999), allowing them to be associated and for the regularity to be detected.

One prediction coming out of this “temporal scaffolding” theory of sleep (Lerner, 2017) is that the detection of temporal regularities following sleep would be more prominent when the time gap between the events constituting the regularity is not too large. This prediction stems from the fact that the effect is bound by the time-compression factor of memory replay (up to ×20 in rats; Euston et al., 2007; the factor is unknown in humans). If the events are too far apart in time, they might not be brought close enough to each other by the time-compression mechanism, making them less likely to be associated. Since the particular length and compression of each replay occurrence can vary across sleep, the shorter the gap between the events, the more likely it is that they will be replayed together, increasing the probability that the regularity to be discovered. In this study, we tested this prediction by evaluating how sleep affects performance in a task that includes temporal regularities with a varying degree of time gaps between predictor and predicted stimuli. We hypothesized that sleep (compared to control) would facilitate the detection of temporal regularities and that this effect will decrease as the time gap increases. In addition, we tested whether performance in the task with and without sleep is related to developing conscious awareness of the temporal regularities governing the stimuli.

## Materials and Methods

### Participants and Design

Eighty-three healthy students from Rutgers University---Newark participated in the study for monetary compensation. Exclusion criteria included personal or family history of sleep problems, neurological or psychiatric disorders, drug or alcohol abuse, and/or intake of medications that might affect sleep. They were instructed to maintain their regular overnight sleep-time schedule and caffeine intake and refrain from alcohol consumption and daytime napping during the study. Participants were randomly assigned to either a Sleep or Wake group, with 40 and 43 participants in each, respectively. Participants in the Sleep group completed the first session of the task at 8 p.m. and the second session at 8 a.m. the following day with a regular sleep at home in between, whereas participants in the Wake group performed the first session at 8 a.m. and the second session at 8 p.m. on the same day and were allowed to carry on with their regular daily activities in between. Participants of each group were further assigned to one of two task settings, differentiating between the type of temporal pattern, or “rule,” used in the experimental task (see below). Twenty-four and 27 participants in the Sleep and Wake groups, respectively, experienced rule A, whereas sixteen participants in each group experienced rule B.^1^ There were no differences in gender, age and education between the Sleep and Wake groups when compared either separately for rule A and rule B, or together (independent *t*-tests, all *p*s > 0.12; see Supplementary Table 1).

### Experimental Task

**FIGURE 1:**
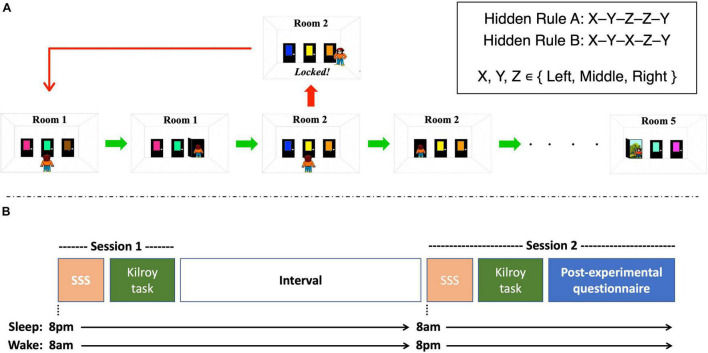
Details on the behavioral experiment. **(A)** Example of a trial in the sequential learning task. Participants guided a character throughout five consecutive rooms, each containing three colored doors. One door led to the next room (or to the exit in the 5th room) and the other two were locked. Choosing a locked door forced participants to start over in room 1. A hidden rule governed the locations of some of the correct doors, the discovery of which allowed choosing the correct door in some of the rooms immediately rather than by trial and error. Two rules were employed, with each participant exposed to only one of the rules. **(B)** Experimental Design. Participants were administered 2 sessions with an interval in between. The sleep group underwent session 1 at the evening and session 2 the following morning and allowed to sleep regularly in between. The wake group underwent session 1 at the morning and session 2 at the evening with no intervening sleep. In each session, participant completed the Stanford Sleepiness Scale (SSS), and 40 trials of the experimental task. In session 2 participants also completed a post-experimental questionnaire examining their conscious awareness of the hidden rule.

We used a novel version of the “Kilroy” sequence learning task (Shohamy et al., 2005; Nagy et al., 2007; Dang et al., 2020), adapted for the purpose of identifying temporal pattern recognition performance. Participants’ objective in each trial was to navigate a cartoon character, Kilroy, through a series of 5 rooms, by choosing the correct door in each room out of three possible choices (Left, Center, Right; see Figure 1A). Two of the doors in each room were locked and one unlocked. The unlocked door led to the next room, or, in the fifth room, to the exit. Choosing a locked door led Kilroy back to room 1, requiring participants to initiate the sequence from the start. Each door was associated with a unique color (15 different colors in total for each trial, randomly assigned to the three doors in each of the five rooms), to help participants remembering the sequence. Critically, unbeknownst to participants, there was a hidden consistency across trials in the location of the correct doors. For rule A, the position of the unlocked doors in the second and third rooms always matched the position of the unlocked doors in the fifth and fourth room, respectively (e.g., Right-Left-Middle-Middle-Left). For rule B, the position of the first room matched the third, and the second room again matched the fifth (e.g., Right-Middle-Right-Left-Middle). Discovering this consistency allowed participants to choose the correct door immediately upon arrival to predictable rooms (rooms 4 and 5 for rule A, rooms 3 and 5 for rule B). The location of the correct door in the remaining, unpredictable rooms was randomized for each trial.

### Procedure

The procedure is depicted in Figure 1B. Upon arrival to the lab in each session, participants completed the Stanford Sleepiness Scale (SSS; Hoddes et al., 1973), to test their sleepiness/alertness levels on a scale of 1–7 (1—fully awake and alert; 7—about to fall asleep). They then sat in a quiet room in the lab and performed the experimental task on a 15” MacBook laptop. The first trial was a practice trial. They then completed 40 experimental trials, each adhering to the hidden rule they were assigned to. Upon completion of the task, participants were released and invited back to the lab 12 h later. The same procedure repeated in the second session, but this time, upon completion of the task, participants were asked to fill in a questionnaire aimed at examining whether they have consciously identified the temporal regularities in the task. They were asked whether they noticed any patterns that helped them perform better, and, if they did, to describe those patterns. They were then given a short generation task, where they were presented with four drawing, each one depicting 5 rooms belonging to a trial in the task, with the correct answers for the unpredictable rooms marked. They were asked to mark which doors are the unlocked ones in the two predictable rooms.

### Statistical Analysis

We evaluated performance in the task based on the probability to correctly choose the right door upon first arrival to each room in a trial. This measure is particularly tailored to estimate whether participants became sensitive to the hidden regularity governing the trials: Consciously identifying the regularity allows choosing the correct door of predictable rooms already on the very first attempt, whereas complete unawareness of the regularity would lead to choosing the correct door on the first attempt in only third of the cases (random chance).

We divided the data from all trials to four quartiles, two for each session (20 trials in each) and computed the average first-attempt accuracy in each quartile for each participant and room (1–5). This resulted in four datapoints for each participant and room, two before the interval and two after the interval. We defined the improvement in sensitivity to the hidden regularities as the difference in first-attempt accuracy between quartile 4 (near the end of the experiment) and quartile 2 (near the end of session 1 just before the interval). Thus, improvement was expected to be 0 for unpredictable rooms, and—potentially—above 0 for predictable rooms. We then defined a new experimental condition with 3 levels, based on the gap between the predictor and predictable room. Gaps could be either zero (room 4 in rule A, predicted by room 3); one (room 3 in rule B, predicted by room 1); or two (room 5 in both rules A and B, predicted by room 2). Data for each gap level was gathered from all relevant participants who experienced that gap. These data were entered to a linear mixed-effects model with fixed effects of Group (Sleep, Wake), Gap (0, 1, 2) and their interaction, and a random intercept. In addition, the individual SSS scores for the second session was entered as covariate to control for its potential effect on the results. Several additional follow-up analyses are described in Results.

To analyze the post-experimental questionnaire, we first determined whether each participant achieved awareness of the hidden regularities, separating between the shorter gaps (0 or 1 for rule A and B, respectively) and the longer gap (2 for both rules). We defined awareness as either: (a) achieving a perfect score in the generation task, or (b) describing the regularity perfectly, or (c) achieving a score of 75% in the generation task while describing the regularity in vague terms (e.g., “I noticed the door location was important”; “I noticed that sometimes the same location repeated room after room”). We then used a chi-square test to compare the groups in terms of their ability to reach awareness to the hidden regularities, as well as *t*-tests to examine whether there were differences in task accuracy between participants who achieved awareness and those who didn’t. Three participants (2 from the Sleep group, 1 from the Wake group) were excluded in this analysis due to not completing the questionnaire. Mixed models analysis was conducted in SPSS 27.0 (IBM); all remaining analysis were conducted in Matlab 2019b (MathWorks).

## Results

We first compared the level of alertness/sleepiness of participants between each group and experimental session, as measured by the SSS scores. Participants in the Sleep group obtained an average of *M* = 2.35 and *M* = 2.77 for Session 1 and 2, respectively, whereas participants in the Wake group had averages of M = 2.23 and *M* = 2.10 for the two sessions (see Supplementary Figure 1). Thus, both groups were alert in both sessions (i.e., SSS score < 3; Berry and Wagner, 2015). Independent *t*-tests showed that there was no difference between the groups in the first session [*t*(81) = 0.572, *p* = 0.57]; however, in the second session, the sleep group showed a somewhat higher levels of sleepiness compared to the wake group [*t*(79) = 2.447, *p* < 0.02)].^2^

**FIGURE 2:**
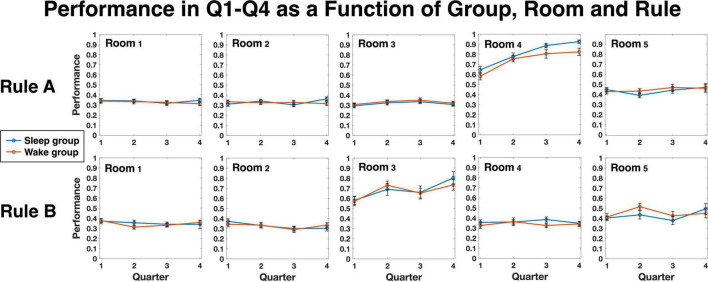
Average performance in the task for each group, room and rule, separated to quartiles 1–4 (Q1–Q4). Each quartile represents 20 trials; quartiles 1, 2 are from Session 1 and quartiles 3, 4 are from Session 2.

**FIGURE 3:**
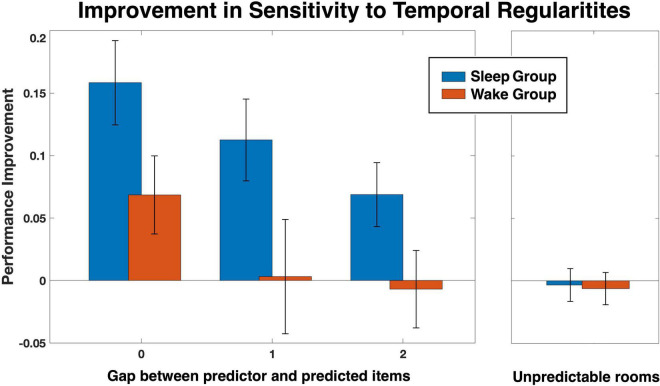
Improvement in sensitivity to the hidden temporal regularities from quartile 2 to quartile 4 for each group as a function of the gap between the predictor and predicted items constituting those regularities. Results are presented alongside the performance for unpredictable rooms, for comparison.

We next turned to examine the sensitivity to hidden regularities, as defined in Methods. Group averages for each quartile and room is depicted in Figure 2, separately for rule A and rule B. As expected, for both groups unpredictable rooms (rooms 1, 2, and 3 for rule A; rooms 1, 2 and 4, for rule B) hovered around 0.33, the random chance level, with little change across quartiles (see also Figure 3, Unpredictable rooms). Predictable rooms (rooms 4, 5 for rule A; rooms 3, 5 for rule B), in contrast, yielded higher-than chance performance early on, which tended to increase with practice. To examine whether sleep preferentially contributed to the increase in performance, we compared the difference score between quartile 4 and quartile 2 as a function of Group and Gap, as described in Methods. The SSS scores of the second session, which were slightly different between the groups, were added as a covariate. A Mixed models analysis showed a significant effect of Group [*F*(1, 91.97) = 8.66, *p* = 0.004], indicating higher improvement for the Sleep compared to the Wake group, and a significant effect of Gap [*F*(2, 108.04) = 3.22, *p* = 0.044], but no interaction (*p* = 0.874). The SSS scores were nonsignificant as well (*p* = 0.832). We therefore reran the analysis without the SSS covariate. Results are presented in Figure 3 and Supplementary Tables 2, 3. Again, we found a significant effect of Group [*F*(1, 98.71) = 9.59, *p* = 0.003], Gap [*F*(2, 112.35) = 3.22, *p* = 0.031], but no interaction (*p* = 0.90). The variance of the random intercept was not significant either (*p* = 0.729; Supplementary Table 4). Bonferroni-corrected pairwise comparisons between gap levels (across groups) showed that the source of the significant gap effect was a performance improvement for gap 0 that was higher than for gap 2 (*p* = 0.026). To further investigate the improvement in sensitivity, we then separately compared each group and gap level to zero, the expected level of improvement when no additional sensitivity to the hidden regularities is obtained over the interval between the sessions. Planned *t*-tests revealed that the improvement was significantly higher than 0 in the sleep group for all three gap levels [*t*(23) = 4.69, *p* = < 0.001, *t*(15) = 3.43, *p* < 0.004, *t*(39) = 2.68, *p* < 0.02, for gaps 0, 1, and 2, respectively], as well as for gap 0 in the Wake group [*t*(26) = 2.19, *p* < 0.04]. No significant effects were found in the Wake group, however, for gaps 1 and 2 (both *p*s > 0.82).

Next, we examined whether sleep affected awareness to the temporal rule. Thirteen participants (32.5%) in the Sleep group acquired awareness to either the short- or the long-gap regularity of the hidden temporal rule in the Sleep group, whereas 16 participants (37.2%) did so in the Wake group. A chi-square test showed that this difference was not significant χ^2^(1, *N* = 80) = 0.13, *p* = 0.72). Similarly, there was no difference between the Sleep and Wake groups when separately examining the percentage of participants becoming aware to the regularity in the short gaps (0 or 1) or the long gap (both *p*s > 0.36).

**FIGURE 4:**
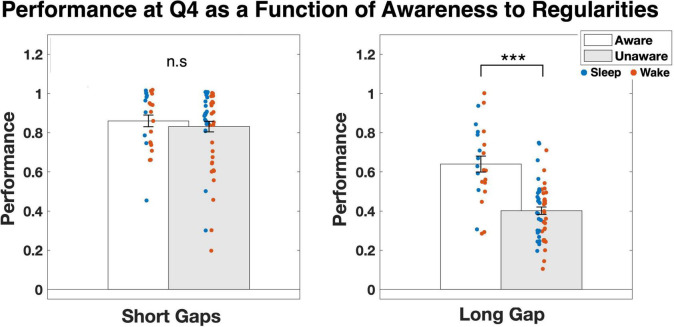
Average performance in quartile 4 for participants who became aware of the hidden regularities compared to participants who did not become aware of the regularities. Results are presented separately for the shorter gaps (0, 1) and the longer gap (2). Individual values for each participant are presented as colorful dots, separately for the Sleep and Wake groups. A small jitter was added to the dots to allow easier visual separation. ***p < 0.001.

We then examined whether there was any relation between performance on the task and awareness to the hidden regularities. To that end, we compared the average first-attempt accuracy in predictable rooms for quartile 4 between participants who became aware of the regularities and those who didn’t. Across all gaps, the average probability was M_aware_ = 0.73 and M_unaware_ = 0.61. An independent *t*-test showed that this effect was highly significant [*t*(78) = 3.906, *p* < 0.001]. We then repeated the analysis for the short and long gaps separately. Whereas for short gaps (0 or 1) there was no difference between the groups (M_aware_ = 0.86 and M_unaware_ = 0.83, *p* = 0.52), for the long gap (2) the difference was, again, highly significant [M_aware_ = 0.64 and M_unaware_ = 0.40; *t*(78) = 6.079, *p* < 0.001; Figure 4]. Hence, whereas high sensitivity to hidden temporal regularities over short gaps could be achieved either with or without conscious awareness of these regularities, high sensitivity to regularities over the long gap was strongly associated with becoming aware of them.

Finally, we examined whether general memory function in the task, independent of temporal pattern recognition, was affected by sleep. To that end, we calculated the probability of making “good choices” in a trial (house) out of the total number of choices made in that trial. A good choice was defined as choosing any door in a room that has not been previously discovered to be locked (i.e., with perfect memory no locked door will ever be chosen more than once). The number of good choices was aggregated over all doors and rooms for each house and divided by the total number of choices for that house, yielding a probability score for that trial. We found that for both groups, memory performance was high across the experiment with averages of M_sleep_ = 0.9700 and M_wake_ = 0.9713. To evaluate the effect of sleep, we computed for each participant the difference in the average probability scores between quartile 4 and 2, similar to the metrics used for assessing temporal patterns learning. These measures were then compared between the two groups using an independent *t*-test. Results showed that there was no difference between the two groups [*t*(81) = 0.398, *p* = 0.69; M_sleep_ = 0.0008 and M_wake_ = –0.0010], indicating that sleep did not improve general memory function in the task. This result held when repeating the analysis using only the rooms that were not predictable by the temporal pattern [rooms 1, 2, and 3 for rule A; rooms 1, 2, and 4 for rule B; *t*(81) = 0.0203, *p* = 0.83].

## Discussion

In this study we tested how sleep affects the discovery of hidden temporal regularities as a function of the time gap between the events constituting those regularities. Our main findings were that, first, independent of sleep, hidden regularities are discovered more easily when gaps are small; and second, that sleep improves the discovery of these regularities for all gaps, and to the same degree. Nevertheless, we also found that for the longer gaps, no additional sensitivity is gained when returning to the task after a wake interval, whereas additional improvement is observed following sleep (Figure 3, Gaps 1, 2). Moreover, conscious awareness to the hidden regularities was associated with performance only for the longest gap, suggesting that while good performance for shorter gaps could have been achieved with or without awareness to the regularity, conscious awareness significantly contributed to performance on the long gap. It is therefore possible that the effect of sleep on performance for this gap could have resulted, at least partially, from gaining explicit insight into the hidden regularity, though more data should be collected to support this conjecture directly. Finally, we found that sleep did not aid general memory function in our task—though, admittedly, that could have resulted from a ceiling effect due to the high memory performance achieved even without sleep.

As a whole, our results are consistent with the temporal scaffolding hypothesis that guided the study, despite not observing an interaction between sleep and gap size. The hypothesis proposes that: (a) when the temporal difference between hidden predictor and predicted events is very short (within Hebbian timescale, i.e., < 200 ms; August and Levy, 1999), good detection of the regularity will be achieved both with and without sleep; (b) with longer temporal differences, wake performance is reduced and thus the effect of sleep will increase; and (c) with still longer temporal differences, sleep performance is reduced as well and thus the effect of sleep will diminish. In other words, the hypothesis suggests that there is a sweet spot for temporal differences where the effect of sleep is maximal. In this study, we did not use very short temporal differences (even a gap of 0 represents about ∼2 s on average between predictor and predicted events) and thus we only expected to see the latter two effects, namely, performance that begins to diminish in the wake condition for earlier gaps, followed by diminishing performance in the sleep condition for larger gaps. While we observed the expected reduction in performance in the Wake group as gaps increased, the effect of sleep, though reducing as well, maintained a similar advantage over wake throughout. The most likely explanation is that we did not use a sufficiently large gap in which the sleep effect is reduced toward 0; alternatively, our statistical power may not have been strong enough to detect subtle effect differences between the gaps. A follow-up study could extend the gap range in both directions, employing both very short and very long gaps, which could potentially capture the full variety of the predicted results.

Our results are also consistent with previous findings from human sleep studies showing that sleep—particularly SWS—facilitates implicit and explicit detection of temporal regularities (e.g., Wagner et al., 2004; Fischer et al., 2006; Yordanova et al., 2008, 2012; Durrant et al., 2011, 2013, 2016; Wilhelm et al., 2013; Lerner and Gluck, 2018). Among those, the strongest evidence for a sleep-induced facilitation in conscious detection of hidden temporal regularities comes from those studies using the Number Reduction Task (NRT; Wagner et al., 2004; Yordanova et al., 2008, 2012) which effectively employed gaps of around 6–7 s on average (Yordanova et al., 2008) between the crucial predictor and predicted events. In our case, the longest gap effectively corresponded to approximately 6–7 s as well (the average time it takes participants to move from room 2 to 5) and was the only condition in which performance was associated with conscious awareness of the hidden regularity. This result is thus consistent with the previous studies and highlights the (still hypothetical) possibility that sleep is especially useful in increasing explicit awareness to unexpected patterns when those patterns span at least several seconds. While we did not find a difference between the Sleep and Wake groups in the prevalence to become aware of the long-gap regularity, it should be noted that we only examined awareness of regularities at the end of the experiment; consequently, we cannot determine whether awareness had been gained before or after the break. Future studies should measure awareness both before and after the interval (cf. Yordanova et al., 2008) to clarify if sleep indeed contributes to the conscious awareness of the long gap regularity, as was found in the NRT paradigm.

Our study has several limitations. First, we modulated sleep using the AM/PM vs. PM/AM procedure, which does not preclude time-of-day-effects stemming from circadian differences between the groups. Previous human studies of memory generalization following sleep differed in their conclusion on whether effects could be attributed to circadian differences or not (e.g., Durrant et al., 2011; Tandoc et al., 2021). We partially controlled for these differences by measuring participants’ sleepiness prior to each session using the SSS. While both groups exhibited alertness (average SSS score < 3; Berry and Wagner, 2015) in each session, the Sleep group did score slightly higher than the Wake group before session 2. However, if anything, that result should have worked against our hypothesis. Moreover, the SSS score did not modulate our results when entered as a covariate in the analysis. A stronger control, however, would include a third group measured at 8 p.m. and then 24 h later (e.g., Tandoc et al., 2021), to verify if the effects of sleep hold when testing times are similar to the Wake group. Another possible control is using a nap paradigm instead of overnight sleep, where both groups experience the task at identical times.

A second limitation of our study is that we did not collect physiological measures of sleep. Such data provides the opportunity to associate a variety of relevant sleep indices (e.g., time spent in SWS, amplitude of slow waves, spindle density, and so on) to the behavioral findings and thus learn more about the mechanisms contributing to the effects. We plan to pursue this line of investigations in future studies.

To conclude, we found that the likelihood to detect hidden temporal regularities embedded in experience decreases as the time gap between the events constituting these regularities increases, with long gaps—but not short gaps—benefiting from explicit awareness to the regularities; and that sleep increases this likelihood for all gaps. Future studies can build on these findings to establish in more detail what sleep-dependent mechanisms contribute to the effects, as well as add additional controls to strengthen the results.

## Data Availability Statement

The raw data supporting the conclusions of this article will be made available by the authors, without undue reservation.

## Ethics Statement

The studies involving human participants were reviewed and approved by the Institutional Review Board in Rutgers University. The patients/participants provided their written informed consent to participate in this study.

## Author Contributions

IL conceived the task, designed the experiment, analyzed the results, and wrote the manuscript. MG oversaw the study. Both authors contributed to the article and approved the submitted version.

## Conflict of Interest

The authors declare that the research was conducted in the absence of any commercial or financial relationships that could be construed as a potential conflict of interest.

## Publisher’s Note

All claims expressed in this article are solely those of the authors and do not necessarily represent those of their affiliated organizations, or those of the publisher, the editors and the reviewers. Any product that may be evaluated in this article, or claim that may be made by its manufacturer, is not guaranteed or endorsed by the publisher.

## Footnotes

1 The number of participants receiving each rule differed due to unforeseen circumstances when running the study; however, as described later, we were not interested in the different rules *per se* but, rather, in specific experimental conditions extracted from both rules.

2 Two participants in the wake group had missing SSS scores for their second session, hence the reduction in degrees of freedom.

## References

[B1] August D. A. Levy W. B. (1999). Temporal sequence compression by an integrate-and-fire model of hippocampal area CA3. *J. Comput. Neurosci.* 6 71–90. 10.1023/a:1008861001091 10193647

[B2] Berry R. B. Wagner M. H. (2015). *Sleep Medicine Pearls*, 3rd Edn. Philadelphia: Elsevier Saunders.

[B3] Dang L. Larson S. P. Gluck M. A. Petok J. R. (2020). Age-related decline in learning deterministic judgment-based sequences. *J. Gerontol. B* 75 961–969. 10.1093/geronb/gby100 30184192 PMC7161369

[B4] Djonlagic I. Rosenfeld A. Shohamy D. Myers C. Gluck M. Stickgold R. (2009). Sleep enhances category learning. *Learn. Mem.* 16 751–755. 10.1101/lm.1634509 19926780 PMC2788212

[B5] Durrant S. J. Cairney S. A. Lewis P. A. (2013). Overnight consolidation aids the transfer of statistical knowledge from the medial temporal lobe to the striatum. *Cerebr. Cortex* 23 2467–2478. 10.1093/cercor/bhs244 22879350

[B6] Durrant S. J. Cairney S. A. Lewis P. A. (2016). Cross-modal transfer of statistical information benefits from sleep. *Cortex* 78 85–99. 10.1016/j.cortex.2016.02.011 27017231

[B7] Durrant S. J. Taylor C. Cairney S. Lewis P. A. (2011). Sleep-dependent consolidation of statistical learning. *Neuropsychologia* 49 1322–1331. 10.1016/j.neuropsychologia.2011.02.015 21335017

[B8] Ellenbogen J. M. Hu P. T. Payne J. D. Titone D. Walker M. P. (2007). Human relational memory requires time and sleep. *Proc. Natl. Acad. Sci. U.S.A.* 104 7723–7728. 10.1073/pnas.0700094104 17449637 PMC1863467

[B9] Euston D. R. Tatsuno M. McNaughton B. L. (2007). Fast-forward playback of recent memory sequences in prefrontal cortex during sleep. *Science* 318 1147–1150. 10.1126/science.1148979 18006749

[B10] Fischer S. Drosopoulos S. Tsen J. Born J. (2006). Implicit learning–explicit knowing: a role for sleep in memory system interaction. *J. Cogn. Neurosci.* 18 311–319. 10.1162/jocn.2006.18.3.311 16602193

[B11] Gomez R. L. Bootzin R. R. Nadel L. (2006). Naps promote abstraction in language-learning infants. *Physiol. Sci.* 17 670–674. 10.1111/j.1467-9280.2006.01764.x 16913948

[B12] Hoddes E. Zarcone V. Smythe H. Phillips R. Dement W. C. (1973). Quantification of sleepiness: a new approach. *Psychophysiology* 10 431–436. 10.1111/j.1469-8986.1973.tb00801.x 4719486

[B13] Lerner I. (2017). “Sleep is for the brain: contemporary computational approaches in the study of sleep and memory and a novel ‘temporal scaffolding’ hypothesis,” in *Computational Models of Brain and Behavior*, ed. Moustafa A. A. (Hoboken, NJ: Wiley), 245–256. 10.1002/9781119159193.ch18

[B14] Lerner I. Gluck M. A. (2018). Individual differences in slow-wave-sleep predict acquisition of full cognitive maps. *Front. Hum. Neurosci.* 12:404. 10.3389/fnhum.2018.00404 30349468 PMC6186812

[B15] Lerner I. Gluck M. A. (2019). Sleep and the extraction of hidden regularities: a systematic review and the importance of temporal rules. *Sleep Med. Rev.* 47 39–50. 10.1016/j.smrv.2019.05.004 31252335 PMC6779511

[B16] Lerner I. Ketz N. A. Jones A. P. Bryant N. B. Robert B. Skorheim S. W. (2019). Transcranial current stimulation during sleep facilitates insight into temporal rules, but does not consolidate memories of individual sequential experiences. *Sci. Rep.* 9 1–17. 10.1038/s41598-018-36107-7 30728363 PMC6365565

[B17] Lewis P. A. Durrant S. J. (2011). Overlapping memory replay during sleep builds cognitive schemata. *Trends Cogn. Sci.* 15 343–351. 10.1016/j.tics.2011.06.004 21764357

[B18] Nagy O. Kelemen O. Benedek G. Myers C. E. Shohamy D. Gluck M. A. (2007). Dopaminergic contribution to cognitive sequence learning. *J. Neural Trans.* 114 607–612. 10.1007/s00702-007-0654-3 17347774

[B19] Plihal W. Born J. (1997). Effects of early and late nocturnal sleep on declarative and procedural memory. *J. Cogn. Neurosci.* 9 534–547. 23968216 10.1162/jocn.1997.9.4.534

[B20] Rasch B. Born J. (2013). About sleep’s role in memory. *Physiol. Rev.* 93 681–766. 23589831 10.1152/physrev.00032.2012 PMC3768102

[B21] Shohamy D. Myers C. E. Grossman S. Sage J. Gluck M. A. (2005). The role of dopamine in cognitive sequence learning: evidence from Parkinson’s disease. *Behav. Brain Res.* 156 191–199. 10.1016/j.bbr.2004.05.023 15582105

[B22] Tandoc M. C. Bayda M. Poskanzer C. Cho E. Cox R. Stickgold R. (2021). Examining the effects of time of day and sleep on generalization. *PLoS One* 16:e0255423. 10.1371/journal.pone.0255423 34339459 PMC8328323

[B23] Wagner U. Gais S. Haider H. Verleger R. Born J. (2004). Sleep inspires insight. *Nature* 427 352–355. 14737168 10.1038/nature02223

[B24] Wilhelm I. Rose M. Imhof K. I. Rasch B. Büchel C. Born J. (2013). The sleeping child outplays the adult’s capacity to convert implicit into explicit knowledge. *Nat. Neurosci.* 16 391–393. 10.1038/nn.3343 23434910

[B25] Yordanova J. Kolev V. Verleger R. Bataghva Z. Born J. Wagner U. (2008). Shifting from implicit to explicit knowledge: different roles of early-and late-night sleep. *Learn. Mem.* 15 508–515. 10.1101/lm.897908 18626095 PMC2505318

[B26] Yordanova J. Kolev V. Wagner U. Born J. Verleger R. (2012). Increased alpha (8–12 Hz) activity during slow wave sleep as a marker for the transition from implicit knowledge to explicit insight. *J. Cogn. Neurosci.* 24 119–132. 10.1162/jocn_a_00097 21812555

