# Supplementary material for: Sleep Facilitates Extraction of Temporal Regularities With Varying Timescales
Source: Front Behav Neurosci. 2022 Mar 25;16:847083. doi: 10.3389/fnbeh.2022.847083 (PMC8990849; doi:10.3389/fnbeh.2022.847083)
Supplement: Supplementary file 1 [file Data_Sheet_1.docx]

Supplementary Material

We supply here a figure and several tables with additional demographic and statistical information, referred to in the main text.

Table S1. Demographic Details of the Groups.

| Group | N (Female) | Age (years) | Education (years) |
| --- | --- | --- | --- |
| Sleep (All) | 40 (21) | 20.20 ± 2.46 | 14.07 ± 1.47 |
| *Rule A* | 24 (13) | 20.25 ± 1.54 | 14.48 ± 1.44 |
| *Rule B* | 16 (8) | 20.12 ± 3.48 | 13.47 ± 1.33 |
| Wake (All) | 43 (26) | 19.72 ± 1.53 | 13.85 ± 1.30 |
| *Rule A* | 27 (18) | 19.63 ± 1.42 | 13.88 ± 1.27 |
| *Rule B* | 16 (8) | 19.87 ± 1.75 | 13.78 ± 1.40 |

Figure S1. Averages Scores on the Stanford Sleepiness Scale (SSS) by Group and Session.


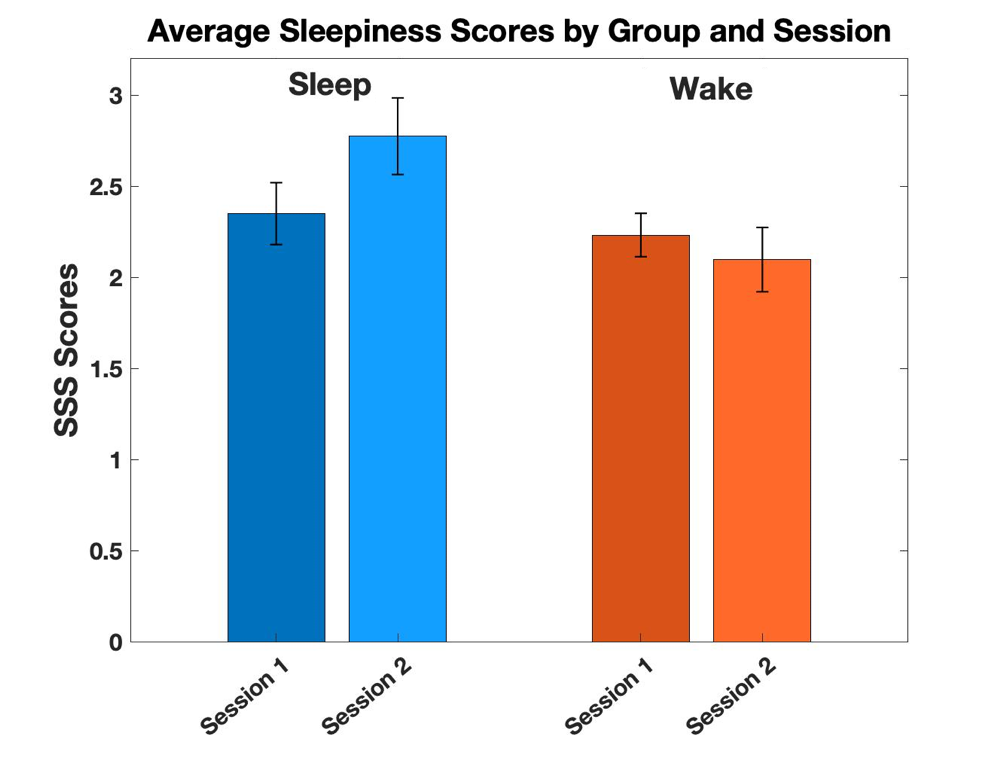


Table S2. Tests of Fixed Effects

| Source | Numerator df | Denominator df | F | Sig. |
| --- | --- | --- | --- | --- |
| Intercept | 1 | 98.711 | 20.967 | .000 |
| Group | 1 | 98.711 | 9.589 | .003 |
| Gap | 2 | 112.350 | 3.595 | .031 |
| Group * Gap | 2 | 112.350 | .105 | .900 |

Table S3. Parameter Estimates of Fixed Effects

|  | | | | | | 95% Confidence Interval | |
| --- | --- | --- | --- | --- | --- | --- | --- |
| Parameter Estimate | | Std. Error | df | t | Sig. | Lower Bound | Upper Bound |
| Intercept | .068750 | .027472 | 159.754 | 2.503 | .013 | .014494 | .123006 |
| [Group=0] | -.075727 | .038168 | 159.754 | -1.984 | .049 | -.151106 | -.000347 |
| [Gap=0] | .089338 | .044188 | 101.147 | 2.022 | .046 | .001683 | .176993 |
| [Gap=1] | .044118 | .050799 | 119.001 | .868 | .387 | -.056469 | .144705 |
| [Group=0] x [Gap=0] | -.015208 | .060967 | 100.166 | -.249 | .804 | -.136162 | .105747 |
| [Group=0] x [Gap=1] | -.031713 | .071502 | 120.461 | -.444 | .658 | -.173275 | .109850 |

Table S4. Parameter Estimates of Covariance Effects

|  | | | | | 95% Confidence Interval | |
| --- | --- | --- | --- | --- | --- | --- |
| Parameter | Estimate | Std. Error | Wald Z | Sig. | Lower Bound | Upper Bound |
| Intercept (Variance) | .001186 | .003420 | .347 | .729 | 4.158084E-6 | .338182 |
